# Supplementary material for: SPP1 Derived from Macrophages Is Associated with a Worse Clinical Course and Chemo-Resistance in Lung Adenocarcinoma
Source: Cancers (Basel). 2022 Sep 8;14(18):4374. doi: 10.3390/cancers14184374 (PMC9496817; doi:10.3390/cancers14184374)

Original Western Blot Picture for Figure 4H

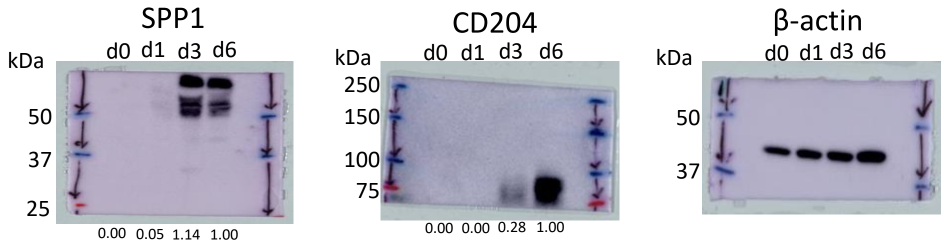

Original Western Blot Picture for Figure 6G

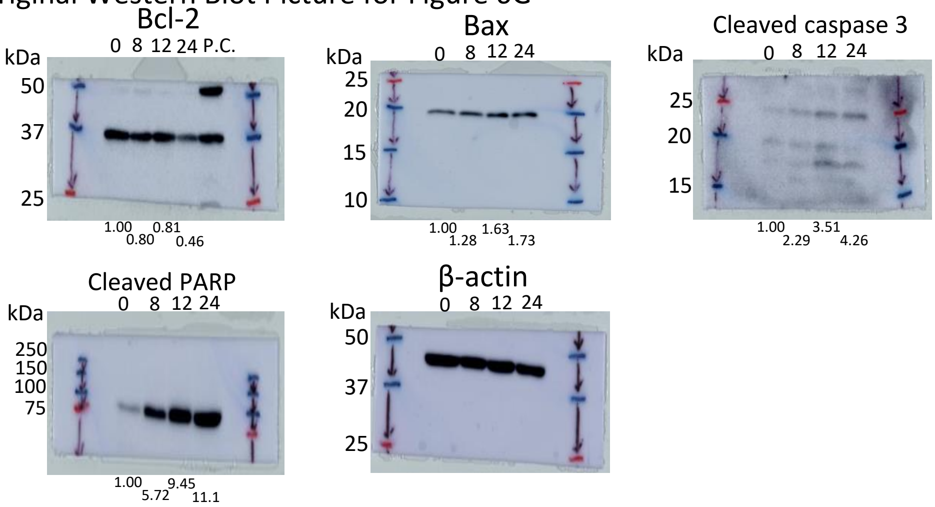

Original Western Blot Picture for Figure 6H

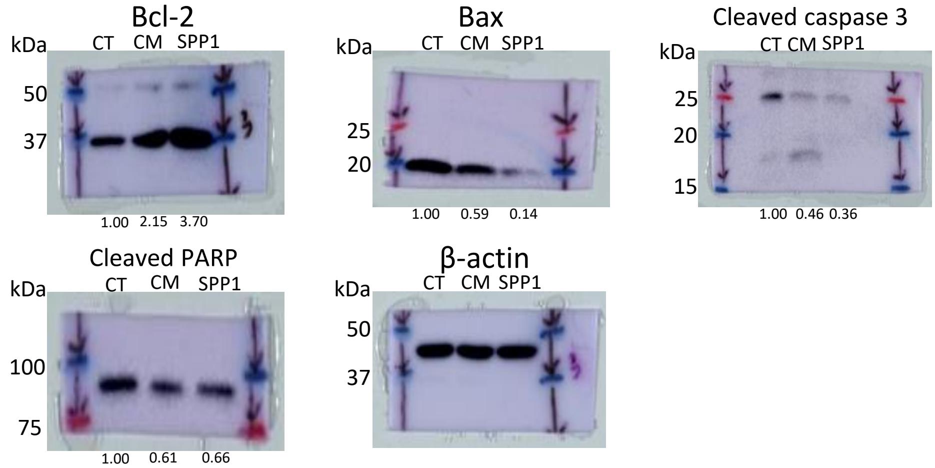

Original Western Blot Picture for Figure 7C

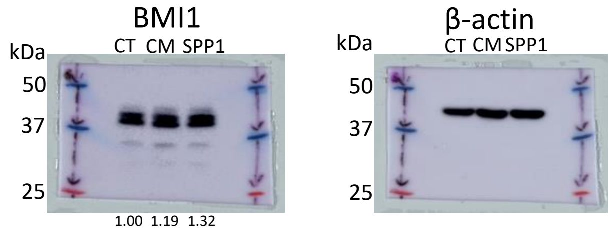

Original Western Blot Picture for Figure 7D

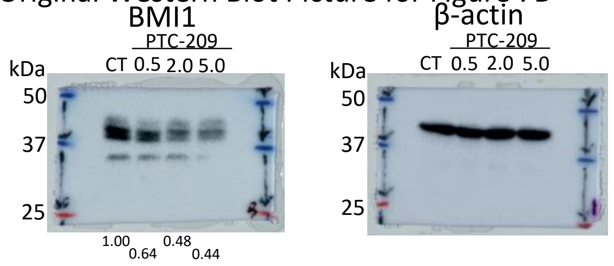

Supplement: Supplementary file 1 [file cancers-14-04374-s001.zip › cancers-1857861-Supplementary File S1.pdf]
