# Supplementary material for: SPP1 Derived from Macrophages Is Associated with a Worse Clinical Course and Chemo-Resistance in Lung Adenocarcinoma
Source: Cancers (Basel). 2022 Sep 8;14(18):4374. doi: 10.3390/cancers14184374 (PMC9496817; doi:10.3390/cancers14184374)
Supplement: Supplementary file 1 [file cancers-14-04374-s001.zip › cancers-1857861-supplementary.pdf]

Supplementary Figure S1

Score 0 vs Score 1 vs Score 2

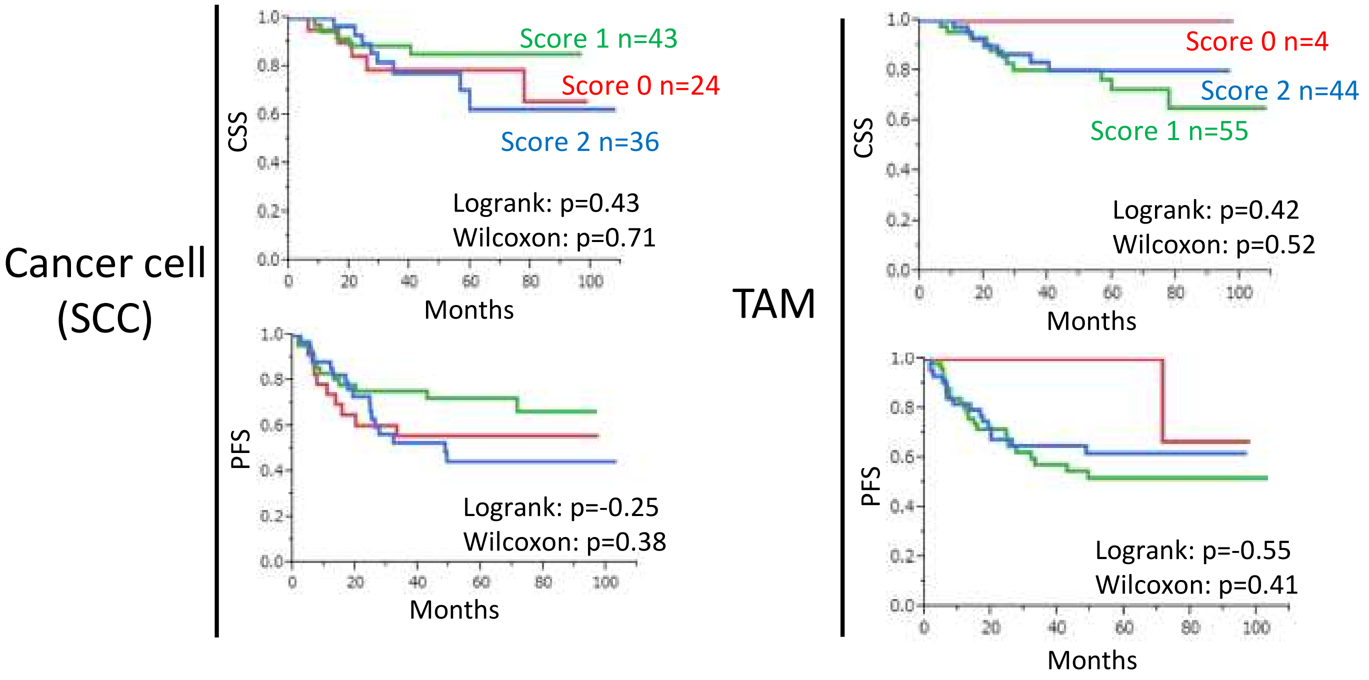

Supplementary Figure S2

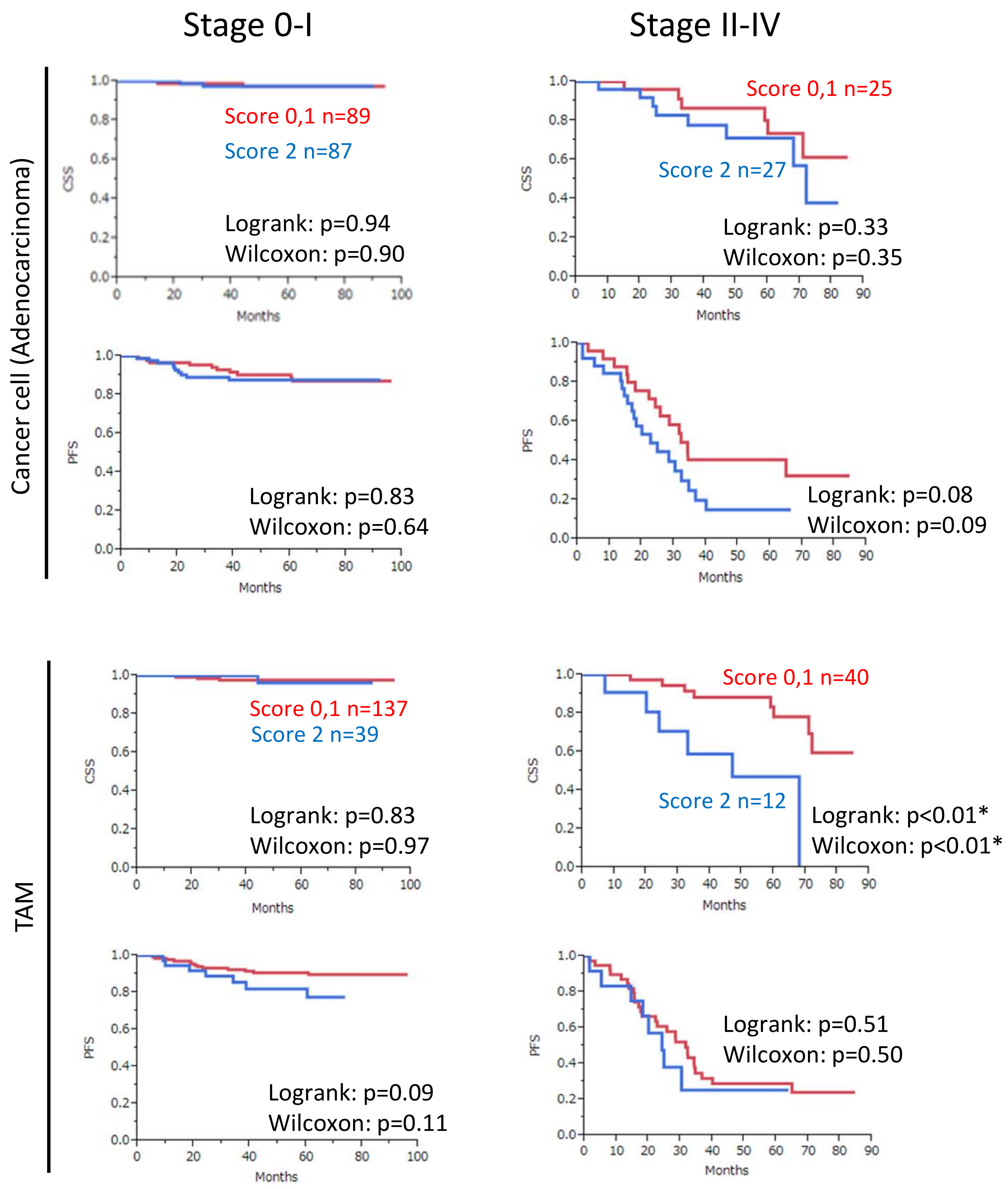

Supplementary Figure S3

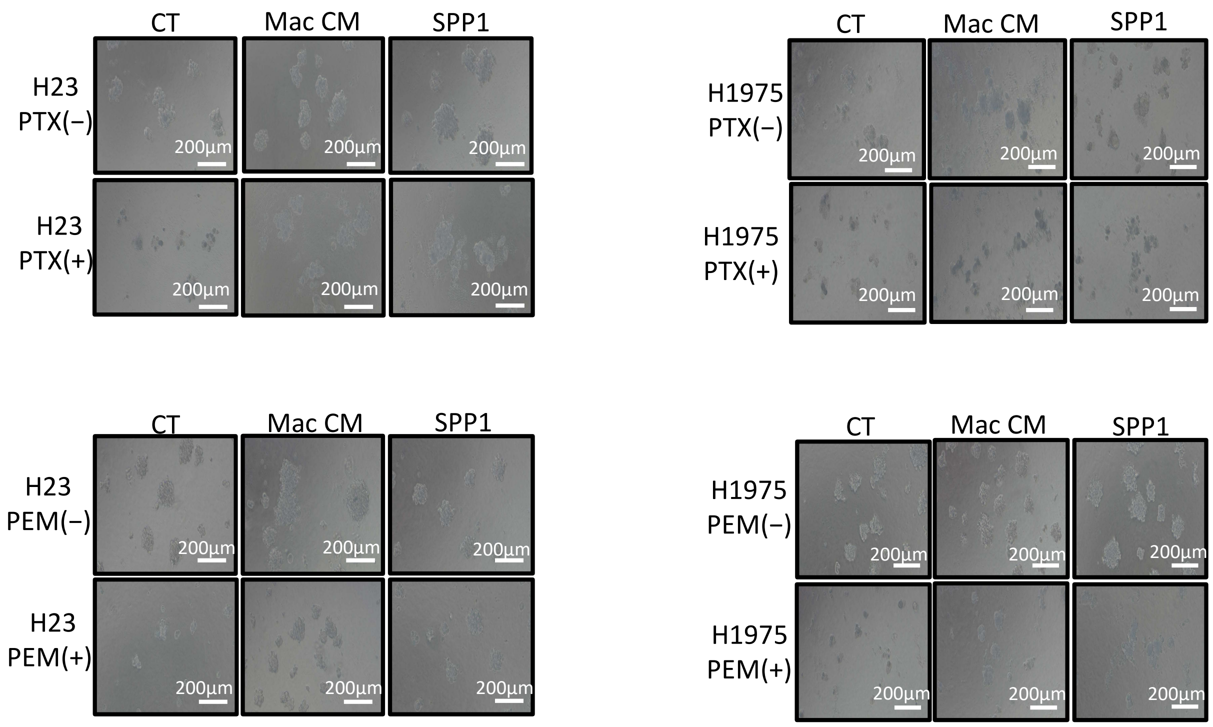

Supplementary Table S1

Target mRNA		Sequence (5’ to 3’)
SPP1	Forward	AGG.AGG.AGG.CAG.AGC.ACA
	Reverse	CTG.GTA.TGG.CAC.AGG.TGA.TG
CD204	Forward	TGG.GAA.CAT.TCT.CAG.ACC.TTG
	Reverse	AAT.CCT.CGT.GGA.CCA.CTT.TC
BMI1	Forward	CTG.CAG.CTC.GCT.TCA.AGA.TG
	Reverse	TTA.GCT.CAG.TGA.TCT.TGA.TTC.TCG.T
β-actin	Forward	ATT.CCT.ATG.TGG.GCG.ACG.AG
	Reverse	AAG.GTG.TGG.TGC.CAG.ATT.TTC

Supplementary Table S2

Cancer-SPP1 expression and clinicopathological factors; adenocarcinoma

		Cancer-SPP1 expression (Low: Score 0,1, High: Score 2)		
		Low	High	p
Age				
	<65	33	34	1.00
	≥65	81	80	
Gender				
	Male	55	59	0.69
	Female	59	55	
Smoking				
	B.I.<600	69	73	0.68
	B.I.≥600	45	41	
pStage				
	0-I	89	87	0.87
	II-VI	25	27	
EGFR mutation				
	negative	60	49	0.20
	positive	49	59	

Chi-square test was performed.

Cancer-SPP1 expression and clinicopathological factors; SCC

		Cancer-SPP1 expression (Low: Score 0,1, High: Score 2)		
		Low	High	p
Age				
	<65	9	11	0.06
	≥65	58	25	
Gender				
	Male	60	33	1.00
	Female	7	3	
Smoking				
	B.I.<600	7	7	0.23
	B.I.≥600	60	29	
pStage				
	0-I	36	16	0.48
	II-VI	31	20	

Chi-square test (Age, pStage) and Fisher’s exact test (Gender, Smoking)was performed.

Supplementary Table S3

	CT	CM	SPP1
BMI1	11.61	14.19	14.6
C18orf32	5.41	10.12	7.57
C19orf71	5.91	6.4	8.23
C1QL4	10.82	17.25	11.65
C4orf48	18.76	27.72	31.83
CDX2	6.59	7.63	8.07
CEBPB	150.27	167.54	184.04
COL1A1	5.92	9.44	7.43
CXCL2	6.92	5.74	8.78
GLIDR	5.38	5.98	7.29
GTF2IP1	13.71	14.32	18.57
HGF	11.59	17.16	10.52
HOXC12	7.48	11.55	9.94
MDP1	13.94	23.92	21.58
MIB2	12.97	16.83	17.54
MRM1	7.74	11.3	10.18
MT1F	7.91	9.33	11.7
NME3	16.76	28.29	22.74
NUDT3	21.19	34.35	28.73
PMS2P2	7.57	9.55	10.08
S100A13	16.27	20.74	20.46
S100A4	9.5	15.34	10.6
SALL2	5.36	7.57	7.12
SNORA4	8.06	31.57	27.71
SNORD35B	10.07	42.06	7.69
SNORD58C	8.12	25.44	31.02
SNORD79	19.34	40.39	44.34
SPIN2B	9.53	12.66	13.33
TNFSF12	6.26	9.07	6.57

Supplementary Table S4

TAM-SPP1 expression and PD-L1 expression of cancer cells in lung adenocarcinoma

	Cancer cells	
	PD-L1 low (<50%)	PD-L1 high (≥50%)
TAM-SPP1 low (Score 0, 1)	167	10
TAM-SPP1 high (Score 2)	47	4

Fisher's exact test was performed (p=0.52).

PD-L1 expression of cancer cells was evaluated based on the percentage of positive staining.

As follows: <low, 50% positive cells; high, ≥50% positive cells.

TAM-SPP1 expression and PD-L1 expression of TAM in lung adenocarcinoma

	TAM	
	PD-L1 low (<50%)	PD-L1 high (≥50%)
TAM-SPP1 low (Score 0, 1)	89	88
TAM-SPP1 high (Score 2)	23	28

Chi-square test was performed (p=0.62).

PD-L1 expression of TAM was evaluated based on the percentage of positive staining.

As follows: <low, 50% positive cells; high, ≥50% positive cells.
